# Alkenes from β-lithiooxyphosphonium ylides generated by trapping α-lithiated terminal epoxides with triphenylphosphine

**DOI:** 10.3762/bjoc.8.219

**Published:** 2012-11-07

**Authors:** David M Hodgson, Rosanne S D Persaud

**Affiliations:** 1 Department of Chemistry, Chemistry Research Laboratory, University of Oxford, Mansfield Road, Oxford, OX1 3TA, UK

**Keywords:** alkenes, epoxides, lithiation, synthetic methods, ylide

## Abstract

Terminal epoxides undergo lithium 2,2,6,6-tetramethylpiperidide-induced α-lithiation and subsequent interception with Ph_3_P to provide a new and direct entry to β-lithiooxyphosphonium ylides. The intermediacy of such an ylide is demonstrated by representative alkene-forming reactions with chloromethyl pivalate, benzaldehyde and CD_3_OD, giving a *Z*-allylic pivalate, a conjugated *E*-allylic alcohol and a partially deuterated terminal alkene, respectively, in modest yields.

## Introduction

**Scheme 1:**
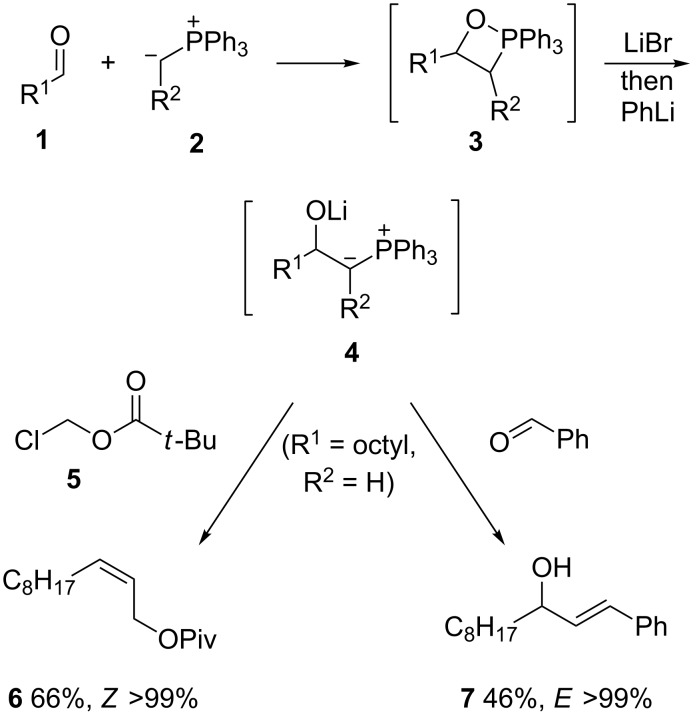
Typical generation of ylide **4** and reaction examples.

β-Lithiooxyphosphonium ylides **4** are useful intermediates in synthesis as they react with a variety of electrophiles to provide a convergent entry to alkenes, often with high regio- and stereocontrol (Scheme 1) [1–9]. These ylide intermediates can be generated by initiating a Wittig reaction between an aldehyde **1** and a phosphorane **2** at low temperature in the presence of lithium salts, which promote ring opening of the initially formed oxaphosphetane **3**, followed by deprotonation typically using PhLi [5].

**Scheme 2:**
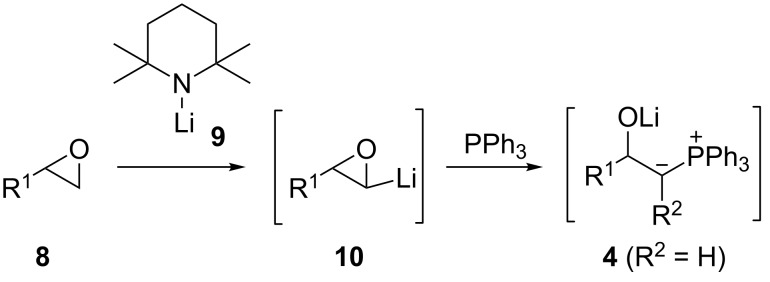
Proposed ylide **4** formation from α-lithiated epoxide **10**.

We recently reported the use of methylenetriphenylphosphorane (**2**) (R^2^ = H) in this chemistry for the synthesis of *Z*-allylic esters such as **6** [8] and conjugated *E*-allylic alcohols such as **7** [9]. β-Lithiooxyphosphonium ylides **4** (R^2^ = H) can also be generated by double deprotonation of β-hydroxy primary phosphonium salts [10–19], where the latter are obtained from Ph_3_P and 1,2-halohydrins [10–16,19] or (in the presence of acid) from terminal epoxides [17–18]. In seeking a more concise way than the above approaches to β-lithiooxyphosphonium ylides **4** (R^2^ = H), we were attracted to the possibility of phosphines intercepting α-lithiated terminal epoxides **10** (Scheme 2) and report here the results of that study. Such carbenoids **10** are unstable, but they can be easily formed from terminal epoxides **8** by using hindered lithium amides, such as lithium tetramethylpiperidide (**9**, LTMP) [20], and have shown synthetically useful carbene reactivity (e.g., cyclopropanation [21–22], dimerization [23–25]). The reaction of carbenes and carbenoids with heteroatom lone pairs is a popular strategy to access ylides [26], although phosphonium ylides for carbonyl-olefination chemistry are usually prepared by deprotonation of phosphonium salts [1–4]. In fact, phosphine trapping of lithium carbenoids followed by carbonyl olefination has been little studied since Seyferth and Wittig independently reported the synthesis of chloro alkenes in modest yields (20–30%) by this route (using CH_2_Cl_2_ and BuLi in the presence of Ph_3_P) over half a century ago [27–31].

## Results and Discussion

**Scheme 3:**
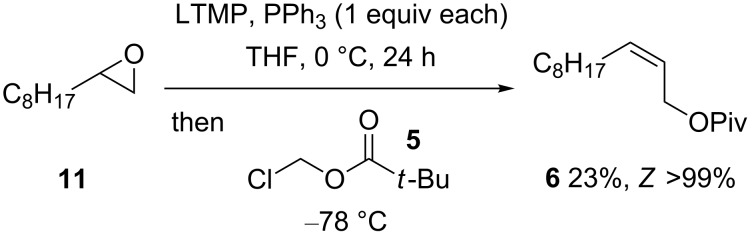
*Z*-Allylic ester **6** from epoxide **11**.

The feasibility of generating and reacting β-lithiooxyphosphonium ylides **4** (R^2^ = H) derived directly from epoxides began with studies to produce allylic ester **6** under LTMP-based conditions for α-lithiation of terminal epoxides [20–22] but with Ph_3_P also present (Scheme 3). Encouragingly, a red-orange colour, which is characteristic of a β-lithiooxyphosphonium ylide [8–9], gradually developed (mixing only LTMP and PPh_3_ in THF at 0 °C for 24 h, gave no colour change from an initial yellow solution), becoming very intense after 3 h, although some epoxide **11** was still present after 24 h (TLC monitoring); the reduced activity of LTMP may be due to phosphine coordination [32]. At this point, following cooling to −78 °C [8], chloromethyl pivalate (**5**) was added, resulting in the isolation of allylic ester **6** (23%). Only the *Z*-isomer of **6** was observed, indicating that stereoselectivity is not altered by this method of β-lithiooxyphosphonium ylide formation. The presence of LiBr (1 equiv) from the start of an otherwise identical reaction made no significant difference to the yield of *Z*-allylic ester **6** (26%), although the presence of such a salt is considered essential for efficient generation of **4** from carbonyl compounds (Scheme 1) [5]; this observation lends support to the notion that the principal role of LiBr is to facilitate oxaphosphetane ring opening to enable subsequent lithiation, and its presence does not significantly influence subsequent reaction steps, at least with this electrophile. While simple phosphoranes (Ph_3_PCH_2_ and Ph_3_PCHMe) are known to react with epoxides (32–68% yields) in the presence of soluble lithium halides [33–34], the homoallylic alcohol, which would arise [35] from any reaction of β-lithiooxyphosphonium ylide and terminal epoxide, was not observed in the present studies; this suggests that the latter ylides are not capable of reacting with terminal epoxides [35], or the presence of LTMP and/or PPh_3_ prevents this reaction from occurring.

The original study on the reaction between LTMP and terminal epoxides in THF showed this to be an efficient way to prepare the corresponding isomerized aldehydes [20] (later established as proceeding through an intermediate TMP enamine) [36–37]. In the present work, neither decanal nor its corresponding TMP enamine were not detected as side-products, and we also established that the presence of LiBr (1 equiv) did not interfere in this isomerization process, giving decanal from epoxide **11** in 65% yield (67% without LiBr) and with no unreacted epoxide observed. The use of shorter reaction times (2–4 h) for the generation of the epoxide-derived ylide **4** (R^2^ = H), including increasing the quantities of LTMP and Ph_3_P (to 3 equiv), or the use of *t*-BuOMe as solvent [21–22], did not lead to improved yields of ester **6**.

**Scheme 4:**
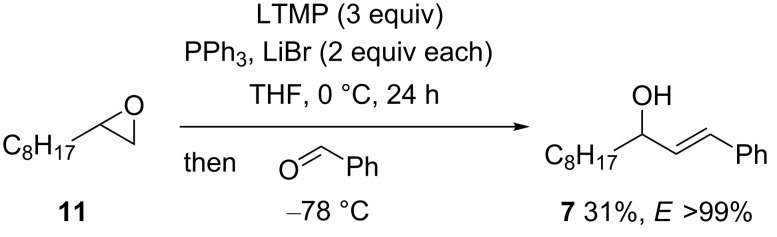
*E*-allylic alcohol **7** from epoxide **11**.

As terminal epoxides are readily available as single enantiomers [38–39], it was considered important to study the possibility of using an aldehyde electrophile with the epoxide-derived ylide. This would provide an entry into allylic alcohols [40], where the epoxide stereocentre is preserved in the product [17–18]. In the event, benzaldehyde was successfully trapped to give *E*-allylic alcohol **7** in up to 33% yield (Scheme 4) by using LTMP (1 equiv), Ph_3_P (5 equiv) and LiBr (2 equiv; 24% yield in the absence of LiBr). Essentially the same yields (31% and 30%) were obtained under otherwise identical conditions but with 2 equiv of Ph_3_P, or with excess LTMP (3 equiv) and Ph_3_P (9 equiv). Other experimental variations (use of sub-stoichiometric TMP (0.25 equiv) [22] or substitution of LiBr by LiCl) did not improve the yield of alcohol **7** (20% and 10%, respectively), whereas substitution of Ph_3_P by Bu_3_P or Cy_3_P did not lead to the orange–red colouration suggestive of ylide formation, and only starting epoxide **11** was observed.

**Scheme 5:**
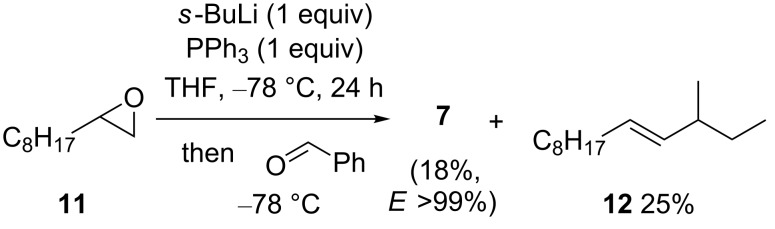
*E*-allylic alcohol **7** and alkene **12** from epoxide **11** by using *s*-BuLi.

We also studied the possibility of generating alcohol **7** from terminal epoxide **11** using an organolithium instead of a hindered lithium amide as the base (Scheme 5). Organolithiums, in particular secondary and tertiary organolithiums, are known to react with terminal epoxides by α-lithiation, although this is typically followed by trapping of the α-lithiated epoxide with a second equivalent of the organolithium and elimination of Li_2_O to give an *E*-alkene (e.g., **12**): a process referred to as reductive alkylation [41]. Also, while PPh_3_ is itself capable of being lithiated–carboxylated (at a meta-position, 6% yield) by using BuLi in Et_2_O [42], this requires significantly higher temperatures (reflux, 46 h) than those applied here. In the event, the use of either *s*-BuLi or *t*-BuLi with epoxide **11** in the presence of Ph_3_P in a variety of solvents (THF, Et_2_O, *t*-BuOMe, toluene) followed by the addition of benzaldehyde was found to give allylic alcohol **7**, albeit in low yields with reductive alkylation always being the dominant reaction pathway, and typically ~30% of epoxide **11** and ~60% Ph_3_P being recovered. The highest yield of allylic alcohol **7** (18%) was obtained by using *s*-BuLi in Et_2_O at −78 °C with a 24 h lithiation time (Scheme 5); lithiation by using other organolithiums (*t*-BuLi, PhLi, BuLi, MeLi), or at higher or lower temperatures (−90 °C or −40 °C), for a longer period (48 h) or in the presence of increased Ph_3_P (2 equiv), or TMEDA (1 equiv) or LiBr (2 equiv) as additives were all less effective.

**Scheme 6:**
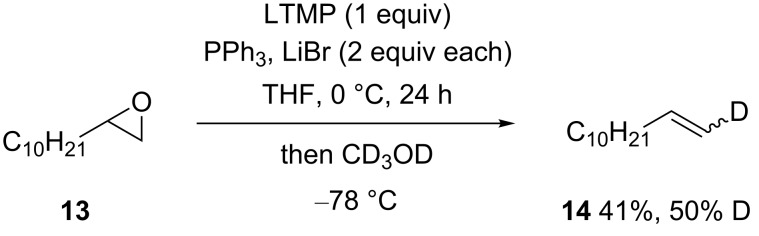
Terminal alkene **14** from epoxide **13**.

The use of a proton (deuterium) source as the electrophile to trap an epoxide-derived ylide prepared by using LTMP was next examined. This was anticipated to provide a base-induced method to deoxygenate epoxides [43], which in the case of deuteration would provide a regiospecific and potentially stereoselective entry to 1-deuterated terminal alkenes [44–45]. Use of a slightly higher molecular weight epoxide, 1,2-epoxydodecane (**13**) to facilitate product isolation, gave dodecene (**14**) (41%, 50% D [46]) after reaction with CD_3_OD (Scheme 6), where the deuterium incorporation was nonstereoselective [44]. Modest deuterium incorporation suggests partial collapse of the intermediate β-lithiooxy ylide occurs under the conditions of its generation, by elimination of Ph_3_PO after or before protonation (e.g., from solvent) and before electrophile addition. Dodecene was also observed as a byproduct in the corresponding reaction of epoxide **13** with benzaldehyde, supporting this hypothesis.

## Conclusion

Among phosphoranes, β-lithiooxyphosphonium ylides occupy a special place, because of their utility in Wittig–Schlosser and SCOOPY-type stereoselective olefination reactions [1–19]. Here we have shown a new and concise method to such valuable intermediates, directly from readily available terminal epoxides. Significantly, the work validates the compatibility of lithium amide and phosphine to generate such ylides, whose intermediacy is demonstrated by representative alkene-forming reactions with chloromethyl pivalate, benzaldehyde and CD_3_OD, giving a *Z*-allylic pivalate, a conjugated *E*-allylic alcohol and a partially deuterated terminal alkene, respectively. High stereochemical control is retained in the *Z*-allylic pivalate and *E*-allylic alcohol syntheses. While the overall yields for the transformations are modest, they stand up to comparison with the earlier methods, given the experimental simplicity and brevity of the current approach.

## Supporting Information

File 1 Preparative details of **6**, **7**, **12** and **14** are reported, together with their spectroscopic data.
