# Supplementary material for: Alkenes from β-lithiooxyphosphonium ylides generated by trapping α-lithiated terminal epoxides with triphenylphosphine
Source: Beilstein J Org Chem. 2012 Nov 7;8:1896–900. doi: 10.3762/bjoc.8.219 (PMC3511028; doi:10.3762/bjoc.8.219)
Supplement: File 1 — Preparative details of 6, 7, 12 and 14 are reported, together with their spectroscopic data. [file Beilstein_J_Org_Chem-08-1896-s001.pdf]

Supporting Information

for

Alkenes from β-lithiooxyphosphonium ylides generated by trapping α-lithiated terminal epoxides with triphenylphosphine

David. M. Hodgson* and Rosanne S. D. Persaud

Address: Department of Chemistry, Chemistry Research Laboratory, University of Oxford, Mansfield Road, Oxford, OX1 3TA, UK

Email: David M. Hodgson - david.hodgson@chem.ox.ac.uk

*Corresponding author

Preparative details of 6, 7, 12 and 14 are reported, together with their spectroscopic data

Contents

1. General details..	S2
2. Synthesis of **6**, **7**, **12** and **14**...	S3
3. ^{1}H and ^{13}C spectra for **6**, **7**, **12** and **14**..	S7
4. References..	S11

1. General details

All reactions were carried out under an atmosphere of nitrogen in flame-dried glassware. THF and Et_2O were degassed and dried over activated alumina under nitrogen [1]. Petrol refers to the fraction of petroleum ether that boils at 30–40 °C. 2,2,6,6-Tetramethylpiperidine was distilled from CaH_2 under reduced pressure (60 °C, 43 mbar). LiBr was made anhydrous by being heated under nitrogen until it melted then cooled and dissolved in THF before being used. PPh_3 was dried overnight (~12 h) under high vacuum (~1 mbar). Benzaldehyde was distilled under reduced pressure (64 °C, 20 mbar) prior to use. Other starting materials were obtained commercially and were used without further purification. TLC analysis was carried out on aluminum-backed plates precoated with silica (60 F_{254}, Merck) and visualised by irradiation under UV light (λ = 254 nm) and by immersion in phosphomolybdic acid (PMA) solution followed by heating. IR spectra were recorded as thin films by using a 1750 Perkin-Elmer Paragon Fourier Transform spectrometer. The strength of absorbance is designated by the following abbreviations: br, s, m, and w, which refer to broad, strong, medium and weak, respectively. ^{1}H and ^{13}C NMR spectra were recorded by using Bruker AV400 and AVC500 spectrometers. Chemical shifts are reported in ppm and referenced to internal residual $CHCl_3$ at 7.27 ppm (^{1}H NMR spectra), and to the central line of the $CDCl_3$ triplet at 77.0 (^{13}C NMR spectra). Coupling constants, *J*, are given in Hz to the nearest 0.1 Hz. ^{13}C NMR data were assigned by standard methods using HSQC and DEPT experiments. *E*/*Z* ratios were determined by ^{1}H NMR analysis of crude products. Mass spectra were obtained by field ionisation (FI; Micromass GCT) or by electrospray ionisation (ESI; LCT Premier Reflectron TOF and Bruker MicroTOF) using tetraoctyl ammonium bromide or sodium dodecyl sulfate as lock mass; values are quoted as ratios of mass/charge (*m*/*z*) in Daltons, and relative intensities of assignable peaks observed are quoted as a percentage value.

2. **Synthesis of 6, 7, 12 and 14**

(*Z*)-Undec-2-en-1-yl pivalate (6)

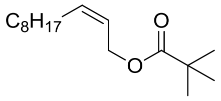

To a solution of 2,2,6,6-tetramethylpiperidine (168 µL, 1.0 mmol, 1.0 equiv) in THF (6 mL) at 0 ºC was added *n*-BuLi (0.40 mL, 2.5 M in hexanes, 1.0 mmol, 1.0 equiv) dropwise under stirring. The mixture was allowed to warm to room temperature over 30 min, during which time a pale yellow solution formed. This solution was then cooled to 0 °C and a solution of PPh_3 (262 mg, 1.0 mmol, 1.0 equiv) and anhydrous LiBr (87 mg, 1.0 mmol, 1.0 equiv) in THF (4 mL) was added dropwise followed immediately by a solution of 1,2-epoxydecane (156 mg, 1.0 mmol, 1.0 equiv) in THF (0.5 mL) slowly dropwise. This solution was then stirred at 0 ºC for 24 h during which time a red-orange colour developed. This mixture was then cooled to −78 ºC and a solution of chloromethyl pivalate (158 mg, 1.05 mmol, 1.05 equiv) in THF (0.5 mL/mmol) added slowly dropwise. The reaction mixture was stirred at −78 ºC for 2 h, then warmed to rt over 1 h and stirring was continued for a further 1 h. The mixture was quenched with saturated aq. NH_4Cl (20 mL), extracted with Et_2O (3 × 15 mL), dried ($MgSO_4$) and evaporated under reduced pressure. Purification of the residue by column chromatography (20% CH_2Cl_2/petrol) gave allylic ester **6** [2] (66 mg, 26%) as a colourless oil; R_f 0.41, 20% CH_2Cl_2/petrol.

IR (neat) /cm^{-1}: 3066s, 3027s, 2957s, 2854m, 1732m, 1730m, 1151s; ^{1}H (500 MHz) δ = 5.66−5.61 (1 H, m, CH=CHCH_2), 5.55−5.50 (1 H, m, =CHCH_2), 4.61 (2 H, dt, *J* = 6.8, 0.60, CH_2OPiv), 2.10 (2 H, app q, *J* = 6.9, CH_2CH=), 1.39−1.23 (m, 12 H, 6 × CH_2), 1.19 (9 H, s, $(CH_3)_3$), 0.88 (3 H, t, *J* = 7.0, CH_3); ^{13}C (125 MHz) δ = 178.5 (C=O), 135.2 (CH_2CH=), 123.6 (=CHCH_2), 60.3 (CH_2O), 38.7 (C), 31.9 (CH_2), 29.4 (2 × CH_2), 29.3 (CH_2), 29.2 (CH_2) 27.6 (CH_2), 27.2 (3 × CH_3), 22.7 (CH_2), 14.1 (CH_3); LRMS (ESI+): 277.20 (75,

M+Na), 413.23 (75), 691.36 (70), 803.45 (100); HRMS (ESI+): 277.2138 calculated for $C_{16}H_{30}O_2Na$; found 277.2143.

(*E*)-1-Phenylundec-1-en-3-ol (7)

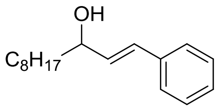

To a solution of 2,2,6,6-tetramethylpiperidine (168 µL, 1.0 mmol, 1.0 equiv) in THF (6 mL) at 0 ºC was added *n*-BuLi (0.40 mL, 2.5 M in hexanes, 1.0 mmol, 1.0 equiv) dropwise under stirring. The mixture was allowed to warm to room temperature over 30 min, during which time a pale yellow solution formed. This solution was then cooled to 0 ºC and a solution of PPh_3 (524 mg, 2.0 mmol, 2 equiv) and anhydrous LiBr (174 mg, 2.0 mmol, 2.0 equiv) in THF (8 mL) was added dropwise followed immediately by a solution of 1,2-epoxydecane (156 mg, 1.0 mmol, 1.0 equiv) in THF (0.5 mL) slowly dropwise. This solution was then stirred at 0 ºC for 24 h during which time a red-orange color developed. This mixture was then cooled to −78 ºC and a solution of benzaldehyde (112 mg, 1.05 mmol, 1.05 equiv) in THF (0.5 mL) added slowly dropwise. The reaction mixture was stirred at −78 ºC for 2 h, then warmed to rt over 1 h and stirring was continued for a further 1 h. The mixture was quenched with saturated aq. NH_4Cl (20 mL), extracted with Et_2O (3 × 15 mL), dried ($MgSO_4$) and evaporated under reduced pressure. Purification of the residue by column chromatography (30% Et_2O/petrol) gave allylic alcohol **7** [3] (77 mg, 31%) as a pale yellow oil; R_f 0.41, 30% Et_2O/petrol; IR (neat) /cm^{-1}: 3351br w, 2924m, 2853m, 1494w, 1450w, 964m, 746s, 691s; ^1H (400 MHz) δ = 7.44−7.24 (5 H, m, Ph), 6.59 (1H, d, *J* = 15.8, CH(OH)CH=CH), 6.26 (1 H, dd, *J* = 15.8, 6.8, CH(OH)CH), 4.30 (1 H, app q, *J* = 6.6, CH(OH)), 1.80 (1 H, br. s, OH), 1.74−1.57 (2 H, m, CH_2CH(OH)), 1.55−1.23 (12 H, m, 6 × CH_2), 0.91 (3 H, t, *J* = 6.8, CH_3); ^{13}C (100 MHz) δ = 136.7 (ArC), 132.6 (CH(OH)CH=CH), 130.1 (CH(OH)CH), 128.5 (2 × ArCH), 127.5 (ArCH), 126.4 (2 × ArCH),

73.1 (CH(OH)), 37.3 (CH(OH)CH_2), 31.8 (CH_2), 29.6 (CH_2), 29.5 (CH_2), 29.2 (CH_2), 25.4 (CH_2), 22.6 (CH_2), 14.1 (CH_3); LRMS (ESI+): 269.19 (80, M+Na), 301.22 (55), 413.32 (55), 515.39 (100); HRMS (ESI+): (M+Na) 269.1876 calculated for $C_{17}H_{26}ONa$; found 269.1868.

***E*-allylic alcohol 7 and alkene 12 from epoxide 11 using *s*-BuLi.**

To a solution of 1,2-epoxydecane (156 mg, 1.0 mmol, 1.0 equiv), PPh_3 (262 mg, 1.0 mmol, 1.0 equiv) and anhydrous LiBr (174 mg, 2.0 mmol, 2.0 equiv) in THF (14 mL) at −78 ºC was added *s*-BuLi (0.77 mL, 1.3 M in cyclohexane/hexane (92/8), 1.0 mmol, 1.0 equiv) very slowly dropwise over ~10 min. The reaction mixture was stirred at −78 ºC for 24 h, and then a solution of benzaldehyde (112 mg, 1.05 mmol, 1.05 equiv) in THF (0.5 mL/mmol) was added slowly dropwise. The reaction mixture was stirred at −78 ºC for 2 h, then warmed to rt over 1 h and stirring was continued for a further 1 h. The mixture was quenched with saturated aq. NH_4Cl (20 mL), extracted with Et_2O (3 × 15 mL), dried ($MgSO_4$) and evaporated under reduced pressure. Purification of the residue by column chromatography (0−30% Et_2O/petrol) gave allylic alcohol **7** (45 mg, 18%) as a pale yellow oil; R_f 0.41, 30% Et_2O/petrol, and alkene **12** [4] (50 mg, 25%) as a colourless oil; R_f 0.87, 15% Et_2O/petrol.

Data for (*E*)-3-methyltridec-4-ene (12)

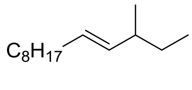

IR (neat) /cm^{-1}: 2958w, 2923m, 2854w, 1457w, 1006w; ^1H (400 MHz) δ = 5.37 (1 H, dt, J = 15.2, 6.3, CH=$CHCH_2$), 5.27 (1 H, dd, J = 15.3, 7.3, CH=$CHCH_2$), 2.07−1.92 (3 H, m, $CHCH_3$, CH=$CHCH_2$), 1.41−1.23 (14 H, m, 7 × CH_2), 0.98 (3 H, d, J = 6.8, $CHCH_3$), 0.94−0.84 (6 H, m, 2 × CH_3); ^{13}C (100 MHz) δ = 136.1 (CH=), 128.7 (=$CHCH_2$), 38.5 (CH), 32.7 (CH_2), 32.0 (CH_2), 30.0 (CH_2), 29.8 (CH_2), 29.6 (CH_2), 29.4 (CH_2), 29.2 (CH_2), 22.8

(CH_2), 20.5 (CH_3), 14.1 (CH_3), 11.8 (CH_3); HRMS (FI+): 196.2191 calculated for $C_{14}H_{28}$; found 196.2194.

1-Deuterio-1-dodecene (14)

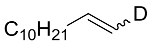

To a solution of 2,2,6,6-tetramethylpiperidine (168 µL, 1.0 mmol, 1.0 equiv) in THF (6 mL) at 0 ºC was added *n*-BuLi (0.40 mL, 2.5 M in hexanes, 1.0 mmol, 1.0 equiv) dropwise with stirring. The mixture was allowed to warm to room temperature over 30 min, during which time a pale yellow solution formed. This solution was then cooled to 0 ºC and a solution of PPh_3 (524 mg, 2.0 mmol, 2 equiv) and anhydrous LiBr (174 mg, 2.0 mmol, 2.0 equiv) in THF (8 mL) was added dropwise followed immediately by a solution of 1,2-epoxydodecane (184 mg, 1.0 mmol, 1.0 equiv) in THF (0.5 mL) slowly dropwise. This solution was then stirred at 0 ºC for 24 h during which time a red-orange color developed. This mixture was then cooled to −78 ºC and neat CD_3OD (162 µL, 4 mmol, 4 equiv) was added dropwise. The reaction mixture was stirred at −78 ºC for 2 h, then warmed to rt over 1 h and stirring was continued for a further 1 h. Saturated aq. NH_4Cl (10 mL) was then added to the mixture, then extracted with Et_2O (3 × 15 mL), dried ($MgSO_4$) and evaporated under reduced pressure. Purification of the residue by column chromatography (0−5% Et_2O/petrol) gave deuterated alkene **14** [5] (69 mg, 41%, 50% D) as a colourless oil; R_f 0.87, 5% Et_2O/petrol.

IR (neat) /cm^{-1}: 2957w, 2923m, 2853w, 1465w, 799w, 724w; ^{1}H (400 MHz) δ = 5.89–5.76 (1 H, m, CH=), 5.05–4.89 (0.99 H, m, =CHD), 2.09–2.00 (2 H, m, CH_2CH=), 1.43–1.18 (16 H, m, 8 × CH_2), 0.89 (3 H, t, *J* = 6.8, CH_3); ^{13}C (100 MHz) δ = 139.0 (=CH), 114.1 (=CH_2), 113.8 (T, $J_{C\text{-}D}$ = 23.6, CDH), 33.8 (CH_2CH=), 32.0 (CH_2), 29.7 (CH_2), 29.6 (CH_2), 29.4 (CH_2), 29.2 (CH_2), 29.0 (CH_2), 22.7 (CH_2), 14.1 (CH_3); HRMS (FI+): 169.1941 calculated for $C_{12}H_{23}D$; found 169.1942.

3. ^{1}H and ^{13}C spectra for 6, 7, 12 and 14

(*Z*)-Undec-2-en-1-yl pivalate (6)

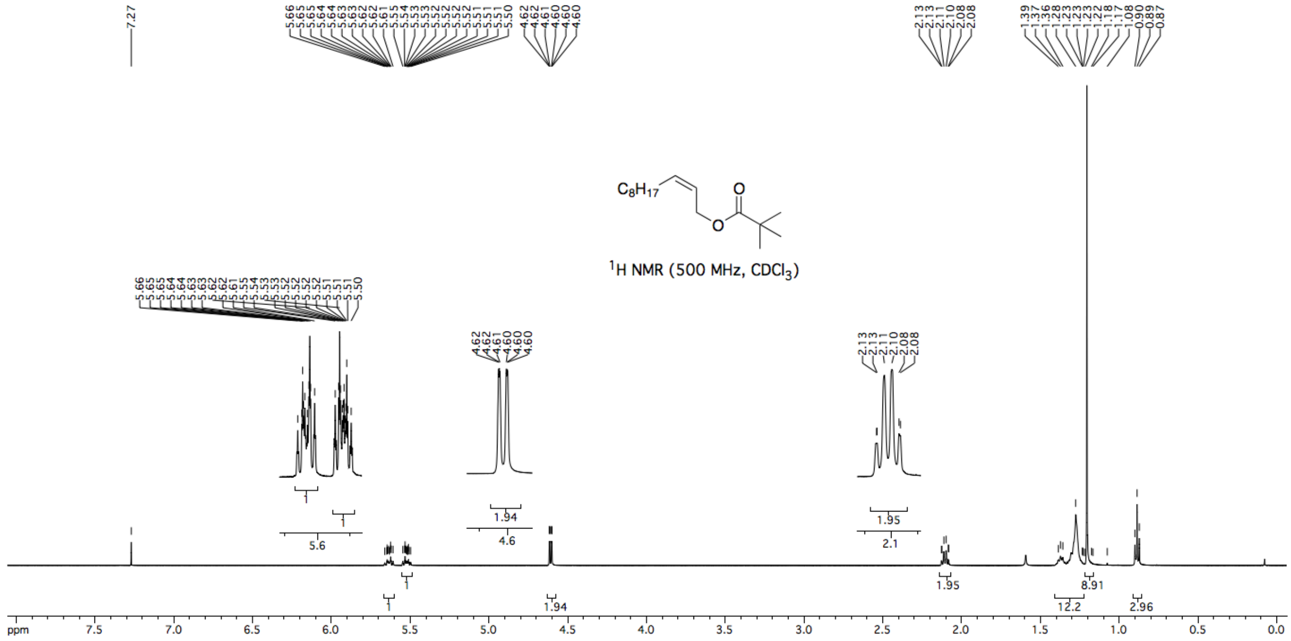

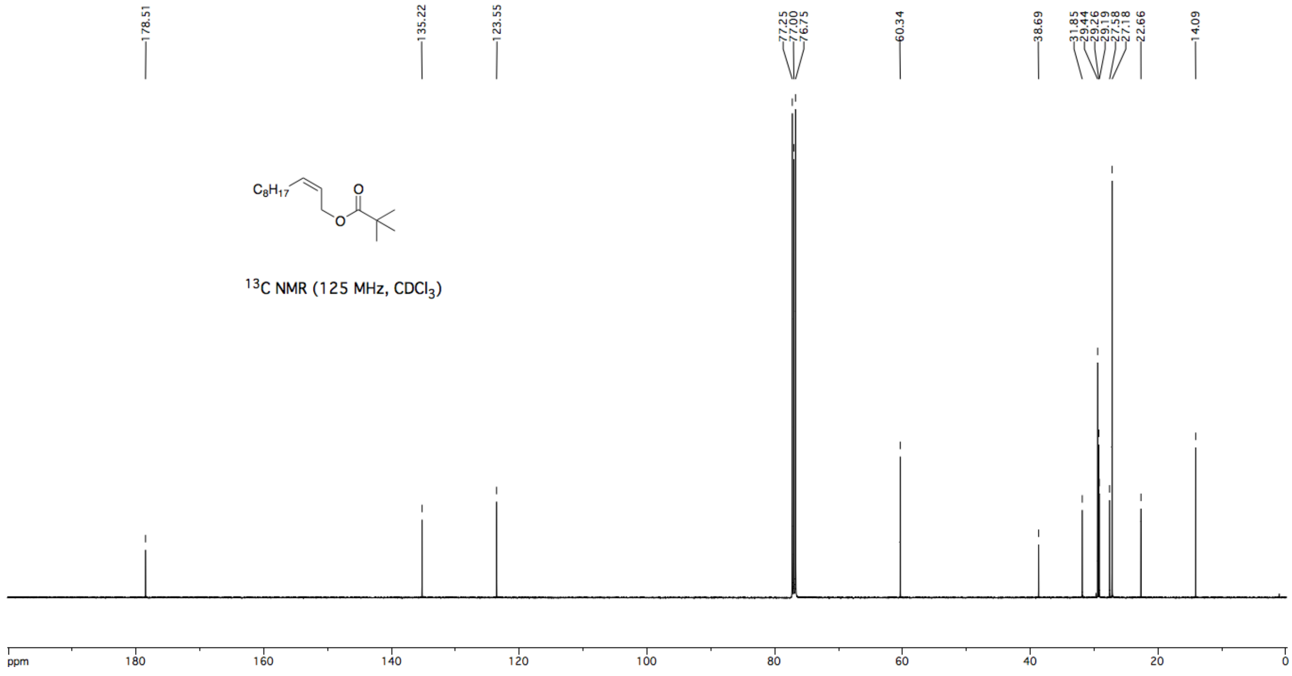

(*E*)-1-Phenylundec-1-en-3-ol (7)

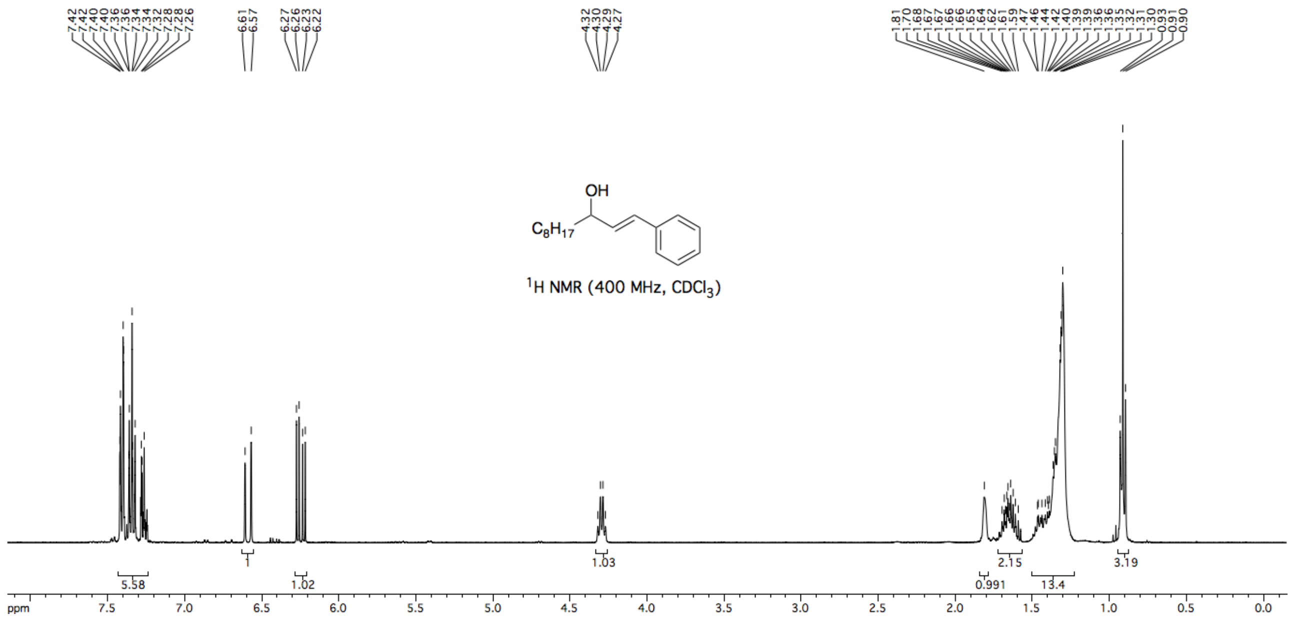

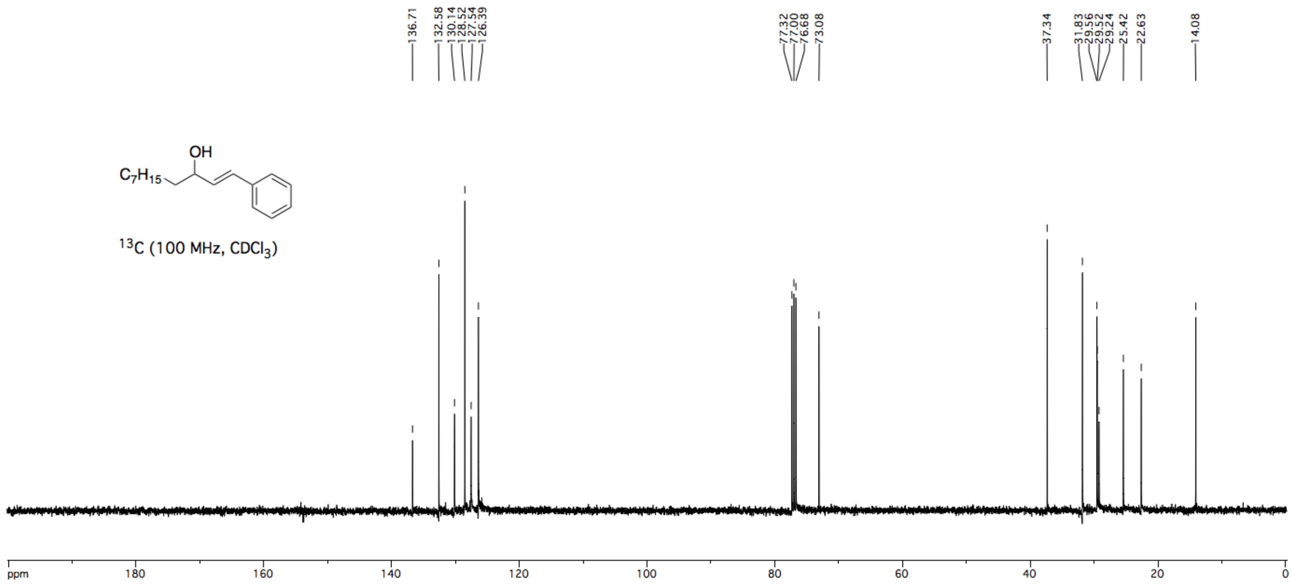

(*E*)-3-Methyltridec-4-ene (12)

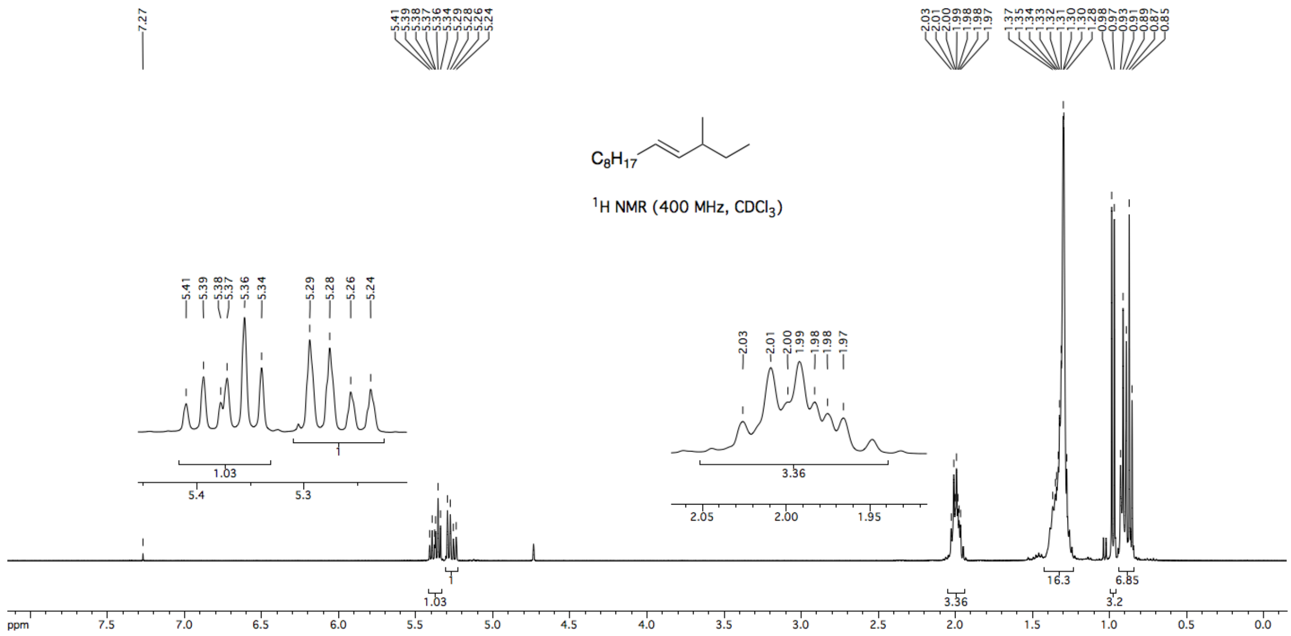

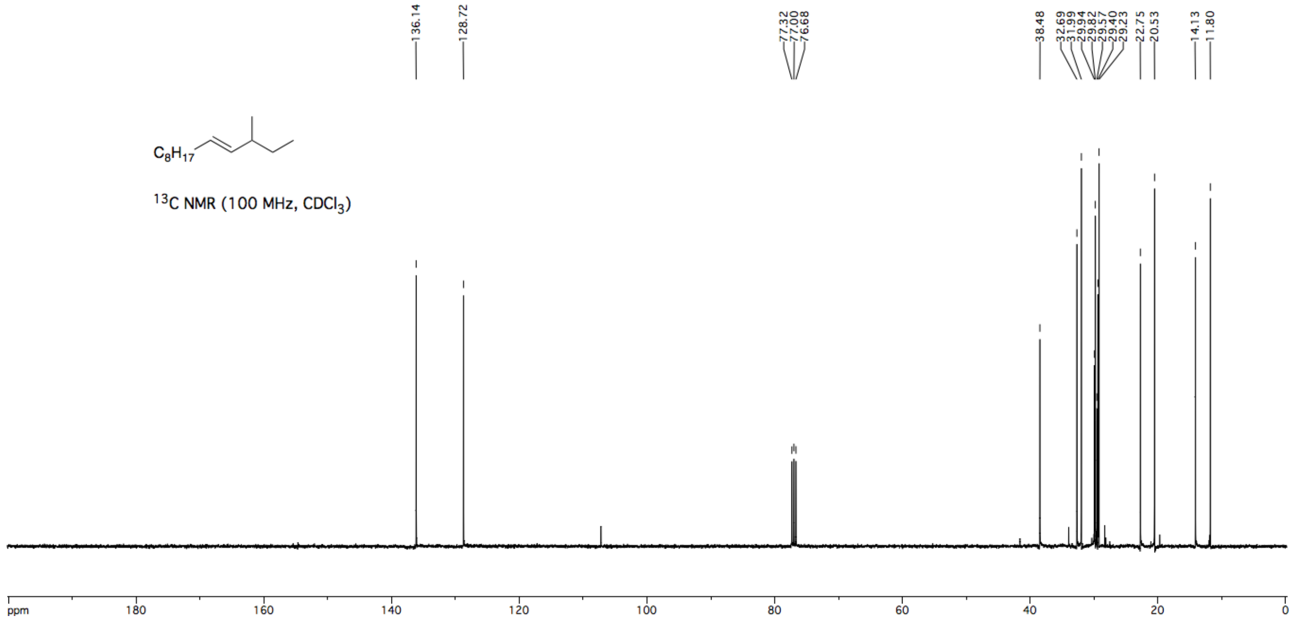

1-Deuterio-decene (14)

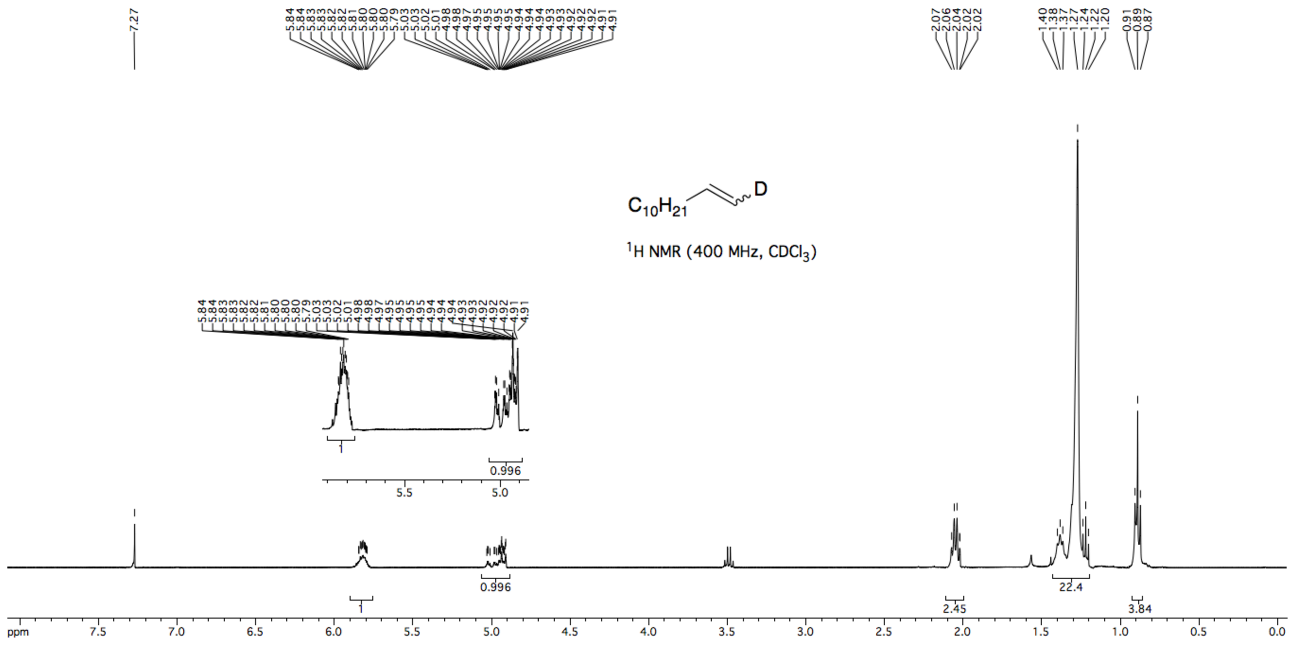

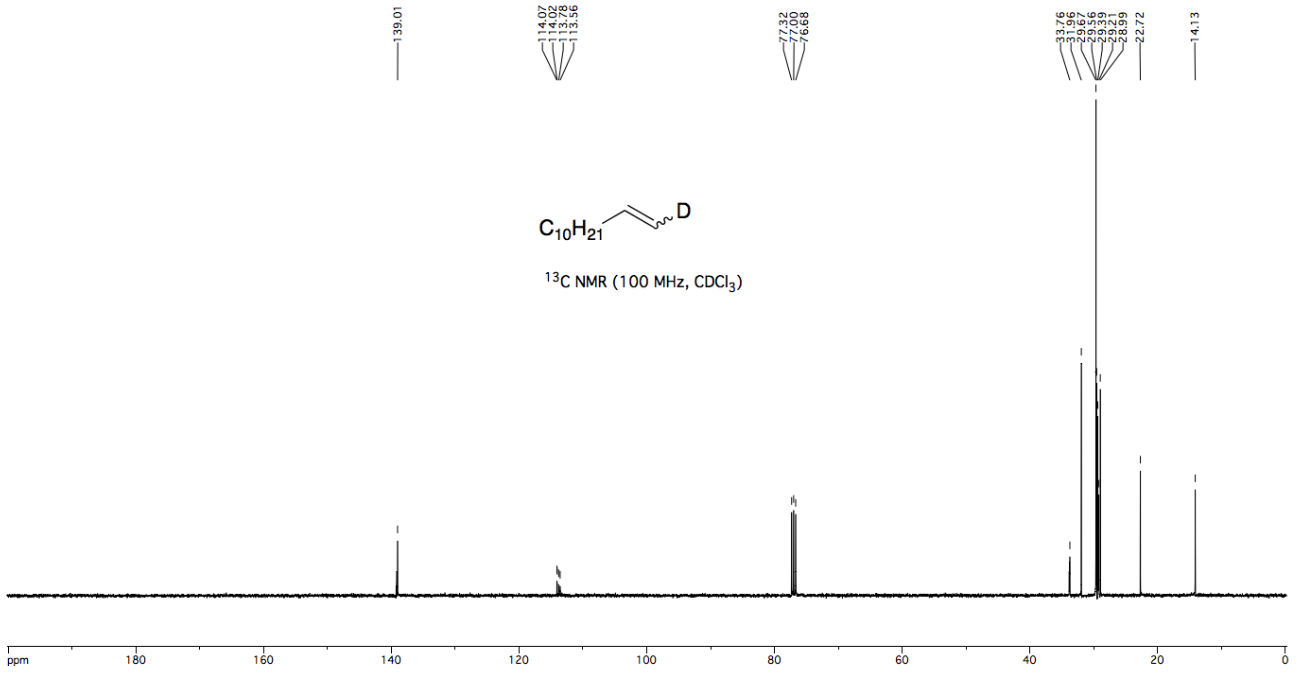

4. References

1. Pangborn, A. B.; Giardello, M. A.; Grubbs, R. H.; Rosen, R. K.; Timmers, F. *J. Organometallics* **1996,** *15,* 1518−1520.
2. Hodgson, D. M.; Arif, T. *Chem. Commun.* **2011,** *47,* 2685−2687.
3. Hodgson, D. M.; Persaud, R. S. D. *Org. Biomol. Chem.,* **2012,** *10,* 7949–7951.
4. Kulinkovich, O. G.; Epstein, O. L.; Isakov, V. E.; Khmel‘nitskaya, E. A. *Synlett* **2001,** 49–52.
5. Oda, H.; Sato, M.; Morizawa, Y.; Oshima, K.; Nozaki, H. *Tetrahedron Lett.* **1983,** *24,* 2877–2880.
